# Flavonoid intake and the risk of age-related cataract in China's Heilongjiang Province

**DOI:** 10.3402/fnr.v59.29564

**Published:** 2015-12-11

**Authors:** Yingna Ma, Weiqi Gao, Kun Wu, Yongping Bao

**Affiliations:** 1 Department of Nutrition and Food Hygiene, Public Health College, Harbin Medical University, Harbin, China; 2 The First Affiliated Hospital, Harbin Medical University, Harbin, China; 3 Department of Nutrition, Norwich Medical School, University of East Anglia, Norwich, UK

**Keywords:** epidemiology, food-frequency questionnaire, diet, crystal aging, quercetin

## Abstract

**Background/objectives:**

Epidemiological evidence suggests that diets rich in flavonoids may reduce the risk of developing age-related cataract (ARC). Flavonoids are widely distributed in foods of plant origin, and the objective of this study was to evaluate retrospectively the association between the intakes of the five flavonoid subclasses and the risk of ARC.

**Subjects/methods:**

A population-based case-control study (249 cases and 66 controls) was carried out in Heilongjiang province, which is located in the northeast of China, and where intakes and availability of fresh vegetables and fruits can be limited. Dietary data gathered by food-frequency questionnaire (FFQ) were used to calculate flavonoid intake. Adjusted odds ratio (OR) and 95% confidence interval (CI) were estimated by logistic regression.

**Results:**

No linear associations between risk of developing ARC and intakes of total dietary flavonoids, anthocyanidins, flavon-3-ol, flavanone, total flavones or total flavonols were found, but quercetin and isorhamnetin intake was inversely associated with ARC risk (OR 11.78, 95% CI: 1.62–85.84, *p*<0.05, and OR 6.99, 95% CI: 1.12–43.44, *p*<0.05, quartile 4 vs. quartile 1, respectively).

**Conclusion:**

As quercetin is contained in many plant foods and isorhamnetin in very few foods, we concluded that higher quercetin intake may be an important dietary factor in the reduction of the risk of ARC.

Age-related cataract (ARC) is the leading cause of poor vision and accounts for almost half of all cases of blindness globally (1). Cataract surgery is regarded as an effective treatment, but it imposes a heavy societal economic burden (2). As the population ages, the number of ARC cases will increase and dietary intervention may be one of the most practical and cost-effective solutions if appropriate risk factors can be identified (3, 4). Oxidation of lens proteins plays an important role in the formation of ARC (5). Flavonoids are effective antioxidants because of their free-radical scavenging properties and because they can chelate metal ions (6). They are metabolic products in fruits and vegetables and they have a number of different phenolic structures (7), some of which may protect the lens against free oxygen radical damage and lipid peroxidation. Individuals with low intakes of fruit and vegetables have been observed to be at increased risk of cataracts (8–10). Flavonoids have not been observed to decrease the risk of ARC related to oxidative damage of the lens in human dietary studies, but the effect has been demonstrated in animal and experimental cell studies. In a mouse model, flavonoids have been shown to increase the activities of antioxidant enzymes (superoxide dismutase and catalase) that can then prevent subsequent selenite-induced cataractogenesis (11–13). Although the relationship between flavonoid intake and risk of ARC remains uncertain, most previous dietary studies have investigated the total intakes of flavonols and flavones and have suggested that flavonoids can have positive effects on other chronic disease risks (14–16). Anthocyanidins, flavan-3-ols, and flavanones were included in our analyses in addition to flavonols and flavones. To show potential differences in the effects of various flavonoids, we also investigated separately the effects of the major flavonols: kaempferol, quercetin, myricetin, isorhamnetin; and the major flavones: apigenin and luteolin.

The purpose of this study was to determine whether intakes of flavonoids were associated with ARC. Heilongjiang province is located in the northeast of China, where the winter is long and the economy is relatively backward. Here, the types and availability of fresh vegetables and fruits are less than in the south of China, and so it was an appropriate research area to explore the relationship between the flavonoid intake and ARC. Our primary hypothesis is that the intake of flavonoids is inversely associated with the incidence of ARC; and a second hypothesis is that the intake of flavonoids is also inversely associated with the incidence of lens opacities.

## Subjects and methods

### Subjects

Between November 2012 and October 2013, 249 ARC patients aged between 50 and 70 years were randomly selected in the Ophthalmic Hospital of Harbin Medical University. Cases were patients diagnosed with ARC admitted to hospital for phacoemulsification surgery. However, patients who had history of other eye diseases or other chronic systemic diseases were excluded except those who had high blood pressure and diabetes (their blood pressure and sugar levels were controlled within the normal ranges). The criteria for ARC were based on a slit lamp observation (17). Subjects were asked to complete a first food-frequency questionnaire (FFQ) on their first or second day in the hospital before surgery. A subsample population of 40 ARC patients was randomly selected and subjected to three consecutive episodes of 24-h dietary record (24-HDR) at home after the surgery. Four weeks after surgery, when the subjects returned to the hospital for check-up, they were asked to complete a second identical FFQ. Meanwhile, 66 controls of the same age with good visual acuity and no eye or systemic diseases were selected at the physical examination center of the same hospital and asked to complete the same FFQ as the ARC study group. This study was conducted according to the guidelines laid down in the Declaration of Helsinki (18), and all procedures involving human subjects were approved by the ethics committee of Harbin Medical University. Written informed consent was obtained from all subjects.

### Measurement of nutrient intakes

The participants responded to a structured interviewer-administered questionnaire that included questions on a wide range of possible risk factors for cataract, including demographic characteristics, lifestyles, medical histories, and family history of cataract. Simultaneously, data about diet were assessed with a validated semi-quantitative FFQ. The FFQ has been used previously in the Heilongjiang province and was suitable for this study (19). Six food groups (staple foods, bean products, meat and eggs, vegetables, fruit, and beverages and milk) which totaled 139 items were included in the FFQ. Among the six food groups, vegetables and fruits are the most important in terms of flavonoid content. Vegetables, included cabbage, rape, Chinese cabbage, cauliflower, broccoli, spinach, celery, lettuce, garden chrysanthemum, sour pickled cabbage, carrots, white radish, eggplant, tomatoes, zucchini, cucumbers, peppers, onions, garlic, green onions, leeks, garlic moss, garlic sprout, taro, yam, ginger, beans, yellow bean sprouts, and mung bean sprout. Fruits included grapes, strawberries, litchi, banana, pineapple, orange, Chinese pear-leaved crab-apple, melon, watermelon, apple, pear, hawthorn, peach, apricot, plum, jujube, and cherry.

In the study, the three consecutive 24-HDR was used to evaluate the validity of the FFQ. The study subjects were requested to write down but not recall as much information as possible about the nature and quantity of the foods that they had consumed during the three consecutive days. The recorded food items were converted into quantities and the intakes of energy and nutrition were calculated for each subject (20). The database used for flavonoid contents of different food items was derived from US Department of Agriculture 2014 statistics (21), which includes data from a number of countries, including China, which was used here. Daily flavonoid intakes were estimated by averaging. The reproducibility of the FFQ was evaluated by comparing data from the same FFQs completed 1 month later. This work was carried out by an experienced research dietitian in order to limit observer differences.

A common unit or portion size for each food was specified, and participants were asked how often on average they had consumed each of the food items listed during the previous year and what was the usual portion size of each item consumed. There were seven possible responses for each food item, ranging from ‘ < 1 time per month to >3 times per day’ and five possible responses for usual portion size of each item, for example, the usual portion size of rice ranging from ‘ < 50 g to >200 g’. The consumption frequency and usual portion size for each food were assigned corresponding weighting coefficients, respectively, with ‘0, 0.1, 0.3, 0.6, 1, 2, 3’ for consumption frequency and ‘0.5, 0.75, 1, 1.5, 2’ for usual portion size (19). The average daily intake of energy and nutrients was calculated by multiplying the consumption frequency of each item by the weighting coefficient for usual size of each food and by its nutrient content per standard size on the basis of China Food Composition 2012 and totaling the nutrient intake for all food items (22). The database used for flavonoid contents of different food items was derived from US Department of Agriculture 2014 (21) and the energy was estimated using the Chinese nutrition calculator software (20). The flavonoid intakes for each food were calculated by multiplying the reported frequency of consumption of specific foods by the flavonoid contents of the specified portion. Intake values were then summed across all foods to obtain the total intake of flavonoid for each individual. Dietary flavonoid density was obtained after adjusting flavonoid intake per 4,186.8 KJ (1,000 kcal) (19).

### Statistical analyses

Statistical Analysis System (SAS9.13) was used for statistical analysis. Case and control demographic and lifestyle factors were summarized. Percentages of measures were calculated. Independent X^2^ tests assessed the significance of any differences. The distributions of energy, energy-adjusted dietary micronutrition, and intakes of flavonoid variables were checked. Median intake and interquartile range were computed for controls and cases before investigating any significant differences by applying the non-parametric Wilcoxon-Rank-Sum test.

Univariate logical regression was applied for the adjustment of energy, and multivariable logistic regression model was applied for the adjustment of energy, age at diagnosis, education, hypertension, diabetes, family income, and waistline:hipline and for intakes of dietary fiber, vitamin B6, vitamin B12, folate, vitamin C, vitamin E, calcium, potassium, copper, and manganese. In secondary analyses for flavones and flavonols, fruit and vegetable intakes were added to the multivariable logistic regression model. Flavonoid, dietary micronutrients, fruit and vegetable intakes were adjusted for total energy using the nutrient residual method before division of subjects into quartiles of intake (lowest to highest). The odds of preventing the development of ARC with increasing intakes of flavonoids were then processed. Multivariate-adjusted OR were adjusted for correlated non-dietary factors, correlated dietary variables, and potential confounders. Factors making a significant contribution were retained in the regression model, including variables with significant correlations with flavonoid intake. The primary analysis was for flavonoids, anthocyanidins, flavan-3-ols, flavanones, flavones, and flavonols; secondary analyses were separately carried out for apigenin, luteolin, kaempferol, myricetin, quercetin, and isorhamnetin (23).

## Results

A total of 249 ARC patients (cases) and 66 controls were recruited. Recruits were from the local population, aged 50–70 years, who had been living in Heilongjiang province for more than 10 years. The socio-demographic, lifestyle characteristics and medical history conditions of the cases and controls included in the study are shown in Table 1. Although cases and controls were all within the range 50–70 years, the average age of cases was older than controls. A higher proportion of cases were diagnosed with hypertension and/or diabetes than controls. Controls were better educated and had more family income than cases. There was no statistical difference between cases and controls in terms of gender, self-assessment for understanding of cataract, long-term residence, exposure to sunlight, smoking, alcohol consumption, tea consumption, physical activity, or body mass index but a higher percentage of controls reported high waistline:hipline ratios compared with cases.

**Table 1:** Demographic characteristics of controls (*n*=66) and cases (*n*=249)

	Controls (%)	Cases (%)	*p*
Age			0.048
50–59	36 (54.55)	102 (40.96)	
60–70	30 (45.45)	147 (59.04)	
Gender			0.550
M	30 (45.45)	103 (41.37)	
F	36 (54.54)	146 (58.63)	
Finished education			<0.001
Primary school	4 (6.06)	118 (47.39)	
High school	32 (48.48)	74 (29.72)	
Undergraduate	30 (45.46)	57 (22.89)	
Long-term residence			0.818
Rural	6 (9.09)	25 (10.04)	
Urban	60 (90.90)	224 (90.96)	
Self-assessment for understanding of cataract			0.454
Unaware	49 (74.24)	173 (69.48)	
Twilight	15 (22.73)	67 (26.91)	
Understand	2 (3.03)	9 (3.61)	
Hypertension			0.001
No	56 (84.85)	160 (64.26)	
Yes	10 (15.15)	89 (35.74)	
Diabetes			<0.001
No	64 (96.97)	184 (73.90)	
Yes	2 (3.03)	65 (26.10)	
Family income (RMB/month)			<0.001
<1,000	1 (1.52)	55 (22.09)	
1,000–2,000	13 (19.69)	80 (32.13)	
>2,000	52 (78.79)	114 (45.78)	
Smoking			0.051
No	55 (83.33)	178 (71.49)	
Yes	11 (16.67)	71 (28.51)	
Alcohol consumption			0.337
No	39 (59.09)	163 (65.46)	
Yes	27 (40.91)	86 (34.54)	
Tea			0.269
Seldom	24 (36.36)	104 (41.77)	
Sometimes	24 (36.36)	94 (37.75)	
Often	18 (27.28)	51 (20.48)	
Physical activity			0.617
Never	30 (45.45)	110 (44.18)	
Occasionally	30 (45.45)	105 (42.17)	
Often	6 (9.10)	34 (13.65)	
Exposure to the sun			0.906
Seldom	29 (43.94)	111 (44.58)	
Sometimes	31 (46.97)	110 (44.18)	
Often	6 (9.09)	28 (11.24)	
BMI			0.208
<18.5	2 (3.03)	7 (2.81)	
18.5–23.9	33 (50.00)	107 (42.97)	
24–27.9	26 (39.39)	102 (40.96)	
>28	5 (7.58)	33 (13.26)	
Waistline:hipline			0.019
M>0.9 or F>0.85	42 (63.64)	118 (47.39)	
M<0.9 or F<0.85	24 (36.36)	131 (52.61)	

M: male; F: female; BMI: body mass index. Statistical significance was accepted with *p*<0.05.

There were significant differences in the average daily intake of energy between cases and controls (*p*<0.001). Consequently, all the nutrient intakes taken from the Chinese food nutrition calculator were adjusted for energy. Median intake and interquartile range of energy and energy-adjusted dietary micronutrition for controls and cases are shown in Table 2. There were significant differences in the average daily intakes of dietary fiber, vitamin B12, folate, potassium, copper, and manganese (*p*<0.001) and also in the average daily intakes vitamin B6, vitamin C, vitamin E, and calcium (*p*<0.05) between cases and controls.

**Table 2:** Energy and dietary micronutrients (energy adjusted) intakes of controls and cases

Intake (per day)	Controls Median (IQR)	Cases Median (IQR)	*p*
Energy (kJ)	8,190.7 (6,813.4–9,120.8)	6,759.1 (5,877.1–8,230.4)	<0.001
Protein (g)	43.24 (39.36–48.83)	43.37 (38.24–48.56)	0.975
Fat (g)	17.48 (14.62–20.24)	17.69 (14.04–20.92)	0.848
Carbohydrate (g)	159.95 (149.91–168.87)	161.57 (149.07–173.16)	0.454
Dietary fiber (g)	12.69 (11.15–14.28)	9.92 (8.89–11.51)	<0.001
Cholesterol (mg)	112.19 (74.16–196.20)	114.48 (71.58–198.76)	0.795
Vitamin A (µg)	468.39 (365.04–579.99)	412.42 (284.73–562.61)	0.056
VitaminB1 (mg)	0.60 (0.53–0.67)	0.59 (0.49–0.69)	0.512
Riboflavin (mg)	0.58 (0.53–0.67)	0.56 (0.46–0.65)	0.068
VitaminB6 (mg)	0.18 (0.15–0.23)	0.23 (0.16–0.28)	0.002
vitaminB12 (µg)	0.03 (0.00–0.09)	0.06 (0.02–0.12)	<0.001
folate (µg)	39.97 (28.09–50.90)	48.89 (33.55–66.93)	<0.001
Niacin acid (mg)	9.65 (8.09–11.33)	9.25 (8.05–11.34)	0.6127
Vitamin C (mg)	93.03 (79.84–106.28)	84.44 (69.05–103.83)	0.038
Vitamin E (mg)	12.42 (10.11–15.49)	11.62 (8.95–14.14)	0.041
Calcium (mg)	399.41 (313.79–478.20)	354.99 (288.60–426.13)	0.032
Phosphorus (mg)	705.68 (642.16–750.34)	706.01 (650.50–751.38)	0.816
Potassium (mg)	1,630.56 (1,483.59–1,986.21)	1,513.74 (1,308.81–1,699.78)	<0.001
Sodium (mg)	376.36 (325.01–493.29)	389.57 (329.27–475.71)	0.809
Magnesium (mg)	218.12 (201.38–250.45)	230.27 (212.83–252.01)	0.081
Iron (mg)	24.88 (17.10–27.35)	14.03 (12.69–15.29)	<0.001
Zinc (mg)	7.19 (6.60–7.74)	7.09 (6.47–7.93)	0.910
Selenium (µg)	23.93 (21.85–29.26)	25.24 (21.62–30.25)	0.517
Copper (mg)	1.33 (1.22–1.49)	1.24 (1.08–1.39)	<0.001
Manganese (mg)	4.65 (4.03–4.99)	3.75 (3.38–4.18)	<0.001
Iodine (µg)	9.57 (7.48–11.96)	9.67 (7.54–12.13)	0.953

Median intake and interquartile range of energy-adjusted dietary flavonoid intake for controls and cases are shown in Table 3. There were significant differences in the average daily intakes of anthocyanidins, flavan-3-ols, flavonols, kaempferol, myricetin, quercetin, isorhamnetin, and flavonoids (*p*<0.001) and also in the average daily intakes of flavones luteolin (*p*<0.05) between cases and controls. Flavonoids include anthocyanidins, flavan-3-ols, flavanones, flavones, and flavonols. Flavones include apigenin and luteolin, and flavonols include kaempferol, myricetin, quercetin, and isorhamnetin.

**Table 3:** Dietary flavonoid intakes (energy adjusted) of controls and cases

	Controls (mg/d) Median (IQR)	Cases (mg/d) Median (IQR)	*p*
Anthocyanidins	17.23 (10.59–22.45)	10.44 (5.51–15.98)	<0.001
Flavan-3-ols	13.3 (10.70–17.14)	10.42 (7.16–15.72)	<0.001
Flavanones	5.11 (2.33–9.09)	6.81 (2.40–10.40)	0.261
Flavones	2.09 (1.63–2.54)	1.79 (1.18–2.37)	0.014
Apigenin	0.45 (0.30–0.69)	0.40 (0.24–0.64)	0.227
Luteolin	1.60 (1.26–1.92)	1.37 (0.91–1.77)	0.004
Flavonols	22.77 (18.86–28.31)	17.07 (12.15–23.03)	<0.001
Kaempferol	8.70 (6.76–11.50)	5.82 (3.89–9.65)	<0.001
Myricetin	0.75 (0.60–0.89)	0.59 (0.41–0.79)	<0.001
Quercetin	11.07 (9.07–13.92)	8.49 (6.20–11.08)	<0.001
Isorhamnetin	1.94 (1.14–2.91)	1.46 (0.72–2.21)	<0.001
Flavonoids	64.92 (53.66–75.61)	51.13 (38.06–64.21)	<0.001

Flavonoids include anthocyanidins, flavan-3-ols, flavanones, flavones, and flavonols. Flavones include apigenin and luteolin and flavonols include kaempferol, myricetin, quercetin, and isorhamnetin.

Energy-adjusted anthocyanidin intake was weakly associated with ARC but this association was not seen after multivariate adjustment. Data for risk of ARC and dietary flavonoid intakes adjusted by energy, age at diagnosis, education, hypertension, diabetes, family income, waistline:hipline, dietary fiber, vitamin B6, vitamin B12, folate, vitamin C, vitamin E, calcium, potassium, copper, manganese, which were significantly different between cases and controls are shown in Table 4. Energy-adjusted apigenin, luteolin, kaempferol, myricetin, quercetin, and isorhamnetin intakes were all associated with ARC. After multivariate adjustment, quercetin and isorhamnetin still showed an association with ARC (Table 5). Multivariate-adjusted quercetin intake was inversely associated with the incidence of ARC.

**Table 4:** Total dietary flavonoid intakes by quartile (Q) of intake and reduction of the risk of age-related cataract (66 controls and 249 cases) (Odds ratios and 95% confidence intervals)

		Energy adjusted			Multivariate adjusted^a^		
			
	Intake (mg/d)	OR	95% CI	*p*	OR	95% CI	*p*
Anthocyanidins							
Q1	<5.94						
Q2	5.94–11.46	1.41	0.56–3.57	0.464	0.86	0.17–4.25	0.855
Q3	11.46–17.95	1.94	0.80–4.71	0.140	0.89	0.19–4.31	0.888
Q4	≥17.95	4.60	2.00–10.58	<0.001	3.81	0.76–19.09	0.104
Flavan-3-ols							
Q1	<7.77						
Q2	7.77–11.11	2.66	0.97–7.33	0.058	1.34	0.24–7.36	0.737
Q3	11.11–16.02	6.32	2.43–16.39	<0.001	4.99	0.88–28.43	0.070
Q4	≥16.02	3.85	1.45–10.26	0.007	7.33	1.00–53.80	0.050
Flavanones							
Q1	<2.35						
Q2	2.35–6.15	1.45	0.70–2.98	0.316	1.21	0.33–4.48	0.776
Q3	6.15–10.37	0.65	0.29–1.48	0.305	0.34	0.08–1.42	0.138
Q4	≥10.37	0.77	0.35–1.7	0.522	0.33	0.07–1.46	0.143
Flavones							
Q1	<1.30						
Q2	1.30–1.90	2.07	0.86–4.97	0.104	1.18	0.24–5.80	0.840
Q3	1.90–2.41	2.47	1.04–5.87	0.040	2.31	0.42–12.65	0.336
Q4	≥2.41	2.78	1.18–6.53	0.019	3.29	0.57–18.91	0.182
Flavonols							
Q1	<12.78						
Q2	12.78–18.92	3.69	1.15–11.88	0.028	1.58	0.31–7.98	0.582
Q3	18.92–24.56	6.36	2.06–19.60	0.001	1.30	0.22–7.86	0.773
Q4	≥24.56	11.1	3.67–33.52	<0.001	0.82	0.1–6.41	0.846
Flavonoids							
Q1	<40.62						
Q2	40.62–54.08	6.35	1.77–22.77	0.005	3.21	0.6–17.15	0.171
Q3	54.08–66.26	7.37	2.08–26.21	0.002	2.26	0.38–13.45	0.371
Q4	≥66.26	14.5	4.19–50.15	<0.001	3.65	0.49–27.29	0.207

a Multivariate adjusted OR for energy, age at diagnosis, education, hypertension, diabetes, family income, waistline:hipline, dietary fiber, vitaminB6, vitaminB12, folate, vitamin C, vitamin E, calcium, potassium, copper, and manganese.

**Table 5:** Total dietary flavones and flavonols intakes by quartile (Q) of intake and reduction of the risk of age-related cataract (66 controls and 249 cases), odds ratios and 95% confidence intervals

		Energy adjusted			Multivariate adjusted^a^		
			
	Intake (mg/d)	OR	95% CI	*p*	OR	95% CI	*p*
Apigenin							
Q1	<0.25						
Q2	0.25–0.40	2.38	1.03–5.49	0.042	1.32	0.32–5.51	0.705
Q3	0.40–0.64	2.07	0.89–4.83	0.092	1.27	0.26–6.12	0.764
Q4	≥0.64	2.00	0.86–4.67	0.107	1.69	0.34–8.38	0.521
Luteolin							
Q1	<0.96						
Q2	0.96–1.4	1.26	0.51–3.10	0.622	0.41	0.07–2.29	0.308
Q3	1.4–1.82	2.54	1.11–5.78	0.027	2.33	0.43–12.71	0.328
Q4	≥1.82	2.62	1.15–6.00	0.022	2.80	0.48–16.29	0.251
Kaempferol							
Q1	<4.16						
Q2	4.16–6.76	3.69	1.15–11.88	0.028	2.80	0.56–13.9	0.208
Q3	6.76–10.11	8.68	2.86–26.39	<0.001	2.33	0.42–12.99	0.333
Q4	≥10.11	8.33	2.73–25.41	<0.001	0.26	0.02–3.05	0.282
Myricetin							
Q1	<0.43						
Q2	0.43–0.63	2.06	0.78–5.42	0.143	3.57	0.61–20.94	0.159
Q3	0.63–0.81	4.15	1.67–10.33	0.002	1.92	0.33–11.06	0.466
Q4	≥0.81	3.70	1.47–9.32	0.006	2.04	0.33–12.68	0.443
Quercetin							
Q1	<6.61						
Q2	6.61–9.08	1.91	0.67–5.46	0.225	2.00	0.32–12.64	0.462
Q3	9.08–11.64	3.80	1.43–10.12	0.008	3.27	0.46–23.41	0.238
Q4	≥11.64	7.50	2.9–19.38	<0.001	11.78	1.62–85.84	0.015
Isorhamnetin							
Q1	<0.87						
Q2	0.87–1.59	2.58	1.05–6.36	0.039	7.96	1.51–41.87	0.014
Q3	1.59–2.37	2.36	0.95–5.85	0.063	1.71	0.27–10.97	0.571
Q4	≥2.37	3.66	1.52–8.81	0.004	6.99	1.12–43.44	0.037

a Multivariate adjusted OR for energy, age at diagnosis, education, hypertension, diabetes, family income, waistline:hipline, dietary fiber, vitaminB6, vitamin B12, folate, vitamin C, vitamin E, calcium, potassium, copper, manganese, and fruit and vegetables.

## Discussion

The genesis of ARC is a multifaceted process derived from the combination of a series of events which induce an array of subtle posttranslational modifications in the lens structural proteins that include aggregation, fragmentation, and precipitation, eventually resulting in opacity (24). Despite the fact that there are a variety of dietary agents that have the potential to prevent ARC formation in animals, a clear recommendation for future interventions in humans to slow down the development of ARC is not yet available. Currently, surgical extraction remains the only approach to cure cataracts (25). In China, surgical resources are limited and are insufficient to provide cost-effective and safe treatment for cataracts for all, and there is a pressing need for inexpensive, non-surgical approaches that can delay the onset of cataracts and thus, as a result, decrease the number of cataract extractions (26). Antioxidant micronutrients have been recommended to protect the population from aging (27, 28). Several clinical studies have pointed to a diminution of ARC incidence after an adequate supply of antioxidants in food. Evidence from epidemiological studies shows that a sufficient intake of fruit and vegetables can reduce the risk of cataract (10, 29). Some other food constituents, such as vitamins, minerals, fiber, and numerous phytochemicals, including flavonoids, may also contribute to the protective effect of fruits and vegetables (4, 30, 31). Recently, considerable attention has been paid to the search for phytochemical therapeutics, but little interest was shown for flavonoids. Flavonoids may exert antioxidant effects because of their ability to act as free-radical scavengers, as hydrogen-donating compounds, as singlet-oxygen quenchers, and as metal ion chelators (32, 33). It is thus meaningful to research the relationships between the risk of ARC and the intake of flavonoids.

ARC most often occurs in individuals over 50 years of age. Epidemiological nutrition studies are considered unsuited for age groups over 70 years; so in this study, we recruited ARC patients aged between 50 and 70 years (34, 35). From the results, we can see that the controls have better education, higher family incomes, greater tendency for hypertension and diabetes, higher energy intake, higher waistline:hipline ratios than the cases, and (by definition) a delay in the onset of ARC. Other previously reported factors (e.g. gender, self-assessment for understanding of cataract, long-term residence, exposure to sun light, smoking, alcohol consumption, tea consumption, physical activity, body mass index) were not found to be significantly different between cases and controls in this study (36–38).

Differences were found between cases and the controls in the intakes of dietary fiber, vitamin B12, folate, potassium, copper, manganese, vitamin B6, vitamin C, vitamin E and calcium, concurring with results reported previously (4, 30, 31). Deficiency of micronutrients may also play a role in the formation of ARC since high intakes of fruit and vegetables containing vitamins C and E have been associated with a significant decrease in the prevalence of cataract (4). However, a recent randomized clinical trial of vitamin E supplementation indicated that it is unlikely to have a beneficial effect on ARC (39). In this study on the relationship between flavonoids and ARC, they have been considered as confounding elements and the data have been adjusted accordingly, that is, in order to nullify these confounding factors, such as vitamins, and other phenolic compounds, such as flavonols and flavones, data were adjusted for the different intakes of fruits and vegetables between cases and controls. Previous studies have shown that quercetin and other flavonoids have protective effects against eye lens opacification (40–42). In this study, associations between dietary intake of five different flavonoid subclasses and ARC risk were assessed in a case–control study in the Heilongjiang province of China. Data from a validated and repetitive FFQ showed that total dietary anthocyanidin, flavan-3-ol, flavanone, flavone, and flavonol intakes were not associated with ARC risk. However, quercetin and isorhamnetin intakes appeared to be related to ARC risk in this population. While quercetin is contained in many plant foods (e.g. tea, most vegetables, most fruits, beans, milk) isorhamnetin is only contained in a small number of plant foods (e.g. onion, green onion, pear, and cherry) which are less commonly consumed. Consequently, more attention was focused on quercetin in this study.

The flavonol, quercetin, is the most widely consumed flavonoid in the human diet, and it is one of the most frequently studied flavonoids (43). In a previous study, quercetin was shown to reduce the risk of cataract formation by modification of multiple pathways pertinent to eye lens opacification, including oxidative stress, non-enzymatic glycation, the polyol pathway, lens calpain proteases, and epithelial cell signaling (44). The data presented here are from the first epidemiological study focusing on the relationship between ARC and diet flavonoids using FFQ in China and confirms a strong association between ARC and quercetin intakes. The antioxidant activity of flavonoids is closely related to their structures. The positioning and the quantity of phenolic hydroxyl groups are important factors in determining their antioxidant activities. Quercetin, 2-(3, 4-dihydroxyphenyl)-3, 5, 7-trihydroxy-flavone, has adjacent phenolic hydroxyl groups on the B-ring which play a major role in its antioxidant activity. Moreover, structure–activity relationships indicated that the combination of –OH groups in the 4-position on the B-ring and in the 7-position on the A-ring bestowed high antioxidant activity; and the addition of the interactions of the three adjacent hydroxyl groups on the B- and A-rings could greatly enhance their antioxidant activities (45). Quercetin not only protects antioxidant enzymes but also enhances the activity of antioxidant enzymes in the body (46). Chethan et al. reported that, of all the flavonoids, quercetin is the most potent inhibitor of aldose reductase (47).

The development of ARC is a slow process, and the effects of lifestyle and environmental factors are difficult to detect in the short term although all recognized factors have been included in this study. While the retrospective nature of the case–control design of the present study makes it potentially susceptible to recall bias, the study has a population-based study design intended to minimize selection biases. The numbers in the study population (249) and the control population (66) are not well matched but this has been considered in the choice of statistical methods used. As a result of the relatively small sample size, multiple testing may also be a potential source of bias. This study's aim was hypothesis generating rather than hypothesis testing. In addition, response rates and the quality of completion of the dietary questionnaires in the cases and controls were comparable. Moreover, it is worth noting that assessment of known risk factors resulted in associations consistent with previous evidence, while reported energy intakes were comparable with previous observations for this population. Additionally, though the food composition database we used was derived from US Department of Agriculture 2014, which is fairly new and comprehensive, the subclass values of some flavonoids in some foods are still absent. In this study, only anthocyanidins, flavan-3-ols, flavanones, flavones, and flavonols were chosen, as they make up the major part of the flavonoid subclass commonly present in plant foods. So the total consumed flavonoid values in the study are representative of the addition of these subclasses and not the total amount consumed. However, it should be noted that the intakes of total flavonoid in the study are lower than the amounts reported from the most recent estimates using the Phenol Explorer database (48). In our study, we have been able to adjust these data to eliminate the items that may also be risk factors for ARC (14).

Heilongjiang province lies at the northeast of China where the weather is cold in winter, and pungent foods, such as onion, hot pepper, and green Chinese onion, were traditionally consumed to defeat the cold. In the countryside, because of shortage of money many people eat the foods they have stored in the autumn, however, in winter, increasing quantities and variety of fresh vegetables have been seen on the table in Heilongjiang. Additionally, in the cities, diabetes patients may follow their doctor's advice to limit the intake of fruit to reduce blood glucose levels. In this study, the main dietary sources of quercetin were from onion which supplied 25%, then apple 12%, hot pepper 11%, and green Chinese onion 7%. On average, less fresh vegetables and fruits were consumed by persons with ARC than persons of the same age without ARC. Onions are a flavonoid -rich food, and the major flavonoid has been identified as quercetin and its conjugates, quercetin-4’-glucoside and quercetin-3, 4’-diglucoside (49). There is good reason to recommend the consumption of quercetin-rich foods such as onion and apple to protect the population from cataract formation. For those preferring not to eat onions or apples, other quercetin-rich fruits and vegetables may be considered as a part of a customized diet designed by a dietician for each ARC patient, which may slow down the formation of ARC. However, as the English proverb states ‘Rome wasn't built in a day’ and eating a quercetin-rich diet lifelong may be the wisest advice. In conclusion, this study demonstrated that dietary quercetin levels are associated with the prevention of ARC, based on epidemiological evidence regarding diet and lens opacity. In another words, quercetin may play a role in prevention of ARC. Future in vivo studies and clinical trials are warranted to assess the benefits of quercetin-rich diets in lowering the risk of ARC.
